# Making gains sustainable: partnering with WASH to stop the transmission of trachoma

**Published:** 2018-06-03

**Authors:** Leah Wohlgemuth, Helen Hamilton, Tim Jesudason

**Affiliations:** 1 Regional Technical Adviser: Sightsavers, London, UK.; 2 Senior Policy Analyst Health and Hygiene: WaterAid, London, UK.; 3 Communications Specialist: International Coalition for Trachoma Control London, UK.

**Partnering with the WASH sector is essential if face washing and environmental improvement components of SAFE are to succeed.**

**Figure:**
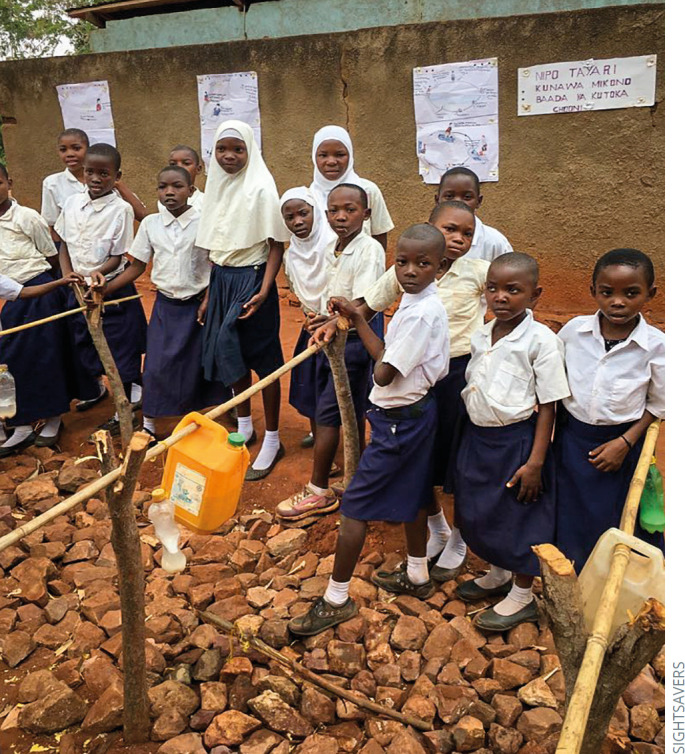
Children learn about hygiene and its link to trachoma and diarrhoea. TANZANIA

There has been impressive progress in recent years to eliminate trachoma. Large numbers of trichiasis operations and antibiotic treatments have contributed to a reduction in the number of people at risk of trachoma, from 325 million in 2011 to 182 million in 2016. In turn, many of the countries that were once among the most endemic, such as Uganda and Tanzania, are now on a path to achieve elimination.

The World Health Organization-endorsed SAFE strategy for trachoma elimination and treatment involves:

- **S**urgery to correct trichiasis
- **A**ntibiotics for *C. trachomatis* infection
- **F**acial cleanliness to reduce transmission
- **E**nvironmental improvements to reduce risk of transmission and infection.

As countries progress towards elimination, new expanded partnerships and strategies to implement facial cleanliness (F) and environmental improvements to prevent the transmission of trachoma (E), usually known as ‘F&E’, are becoming increasingly important to sustain the progress being made.

Through two major partnership initiatives, The Queen Elizabeth Diamond Jubilee Trust's Trachoma Initiative and the UK's Department for International Development (DFID) SAFE Program, new efforts are being made to partner with the water, sanitation and hygiene (WASH) sectors. The International Coalition for Trachoma Control (ICTC) ‘All you need for F&E’ toolkit is providing programme managers with leading practices about partnering with the WASH sector to conduct joint planning and coordination of F&E activities. Additionally, through the Neglected Tropical Disease NGO Network (NNN) WASH Working Group, a cross-sectoral partnership of key NTD and WASH stakeholders, priorities are being identified so that gaps in the implementation of F&E work can be filled.

**Figure:**
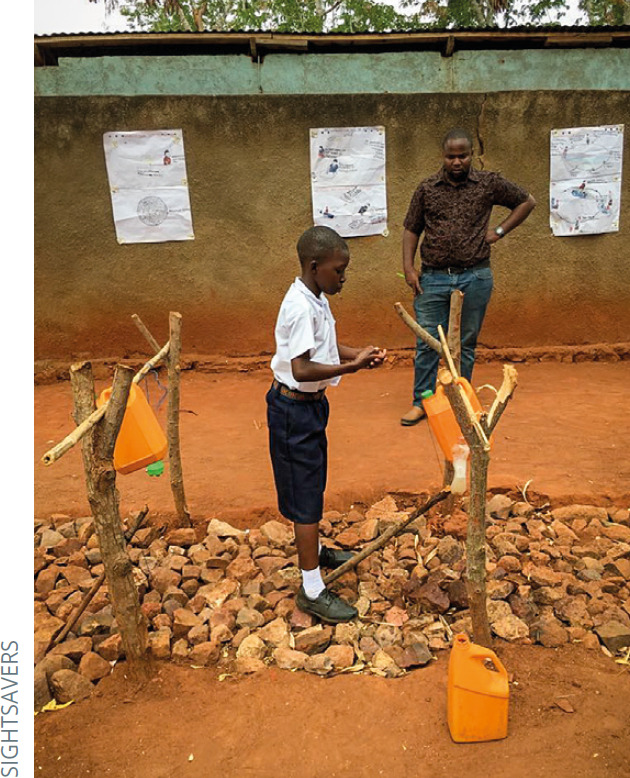
Learning about hygiene. TANZANIA

In Uganda, messages that promote facial cleanliness are being directly integrated into community programmes led by WASH stakeholders. Incorporating facial cleanliness promotion into existing structures, such as the Mother Care Group (which already educates mothers about hand washing and sanitation) avoids duplication of efforts. Moreover, working alongside WASH organisations and community groups that have an established trusted presence in the community increases the uptake of healthy behaviours, since community members are already engaged with the work being done.

Similarly, in Tanzania, partners are working with district health officers to involve community and religious leaders in community-led total sanitation and hygiene (CLTS-H) projects. By equipping leaders with information about trachoma prevention, communities can mobilise and take ownership over their own health behaviour. CLTS-H is recognised as being a crucial element in the uptake of sustained healthy behaviours, and it can be implemented with limited resources.

In Uganda, partners are also working with health authorities to revise national and school sanitation guidelines to include F&E education for the prevention of trachoma. This increases the reach of hygiene messages and ensures sustainability even after trachoma programmes reach completion. This also strengthens national health programmes and systems, which are an important building block for the achievement of Universal Health Coverage.

Despite significant progress, the trachoma community on its own cannot implement the F&E components of SAFE on a large enough scale. New partnerships are needed at all levels to ensure F&E interventions will reach all communities and be sustained. Scaling up WASH activities will contribute to the elimination of other NTDs and government health priorities such as diarrhoea, a major cause of childhood mortality. Partnering with the WASH sectors and scaling up activities will not only put the trachoma community on the path to achieve global elimination, but also help the world achieve the 2030 Sustainable Development Goals, with no one left behind.

